# Barriers to SARS-CoV-2 Testing among U.S. Employers in the COVID-19 Pandemic: A Qualitative Analysis Conducted January through April 2021

**DOI:** 10.3390/ijerph191811805

**Published:** 2022-09-19

**Authors:** Alberto J. Caban-Martinez, Claudia Parvanta, Naciely Cabral, Cynthia K. Ball, Adrienne Eastlake, Jeffrey L. Levin, Kevin Moore, Dalia Nessim, Ernie Stracener, Matthew S. Thiese, Paul A. Schulte

**Affiliations:** 1 Department of Public Health Sciences, Leonard M. Miller School of Medicine, University of Miami, Miami, FL 33136, USA; 2 College of Public Health, University of South Florida, Tampa, FL 33612, USA; 3 Department of Occupational and Environmental Medicine, School of Medicine, University of Texas at Tyler, Tyler, TX 75708, USA; 4 National Institute for Occupational Safety and Health, Cincinnati, OH 45226, USA; 5 Department of Biosystems and Agricultural Engineering, Ferguson College of Agriculture and the College of Engineering, Architecture and Technology, Oklahoma State University, Stillwater, OK 74078, USA; 6 Wisconsin State Laboratory of Hygiene, Madison, WI 53706, USA; 7 Department of Family & Preventive Medicine, School of Medicine, University of Utah, Salt Lake City, UT 84108, USA; 8 Advance Technologies and Laboratories International, Gaithersburg, MD 20878, USA

**Keywords:** COVID-19, SARS-CoV-2, companies, organizations, testing, barriers

## Abstract

During the first year of the COVID-19 pandemic, U.S. companies were seeking ways to support their employees to return to the workplace. Nonetheless, the development of strategies to support the access, use, and interpretation of SARS-CoV-2 testing was challenging. In the present study, we explore, from the perspective of owners and company leadership, the barriers to SARS-CoV-2 testing among U.S. companies. Key informant interviews with company representatives were conducted during January–April 2021 about SARS-CoV-2 testing. A pre-interview survey assessed respondent socio-demographic and organizational characteristics. Interview sessions were transcribed, coded, and analyzed using MaxQDA. A total of twenty interviews were completed with at least two interviews conducted in each major U.S. industry sector. Ninety percent of participants represented companies in business >10 years, comprising both small and large workforces. Using a grounded theory approach, six themes emerged: (1) access to and knowledge of SARS-CoV-2 tests; (2) strategies for symptomatic and asymptomatic testing of workers; (3) type/availability of personal protective equipment to mitigate coronavirus exposures; (4) return-to-work policies; (5) guidance and communication of SARS-CoV-2 Testing; and (6) use of contact tracing and SARS-CoV-2 vaccination. Various modifiable and non-modifiable challenges for SARS-CoV-2 testing among U.S. companies were identified and can inform work-related SARS-CoV-2 testing strategies.

## 1. Introduction

Following lockdowns during the first year of the COVID-19 pandemic, U.S. companies were increasingly asking their employees to return to the workplace. Nonetheless, the development of strategies to support the access, use, and interpretation of SARS-CoV-2 testing was challenging [1]. As businesses were recovering from the pandemic’s economic turmoil while planning to ensure the safety of workers, many were confronted with the need to determine how to use and access SARS-CoV-2 testing for their employees [2,3]. Small businesses, which typically have fewer financial resources than large firms, were grappling with the logistics, costs, and privacy implications of testing their workers for SARS-CoV-2 [4,5]. Businesses for which an outbreak among employees could be extremely costly, possibly halting operations, were most frequently seeking out SARS-CoV-2 testing options [6,7]. Some employers were worried that information on infection discovered through testing could make them liable to lawsuits from workers or customers [7]. While the federal government made funds available to small businesses through the Paycheck Protection Program loans, most of those funds had to be used for specific purposes such as payroll [8,9]. That meant that SARS-CoV-2 testing and other safety precautions were an added financial burden, while also a likely source of confusion. How businesses went about offering SARS-CoV-2 testing to their employees early in the COVID-19 pandemic is unclear.

In April 2020, the U.S. White House provided broad guidance as to whether and how frequently employers should test workers for SARS-CoV-2 [10]. This guidance provided critical industries and sectors, such as healthcare, public safety, and food processing, with counsel on implementing SARS-CoV-2 testing strategies [11]. The guidelines at the time advised all businesses of all workforce sizes to conduct daily health checks, implement physical distancing practices, and encourage employees to wear face masks. It was unclear at the time to what extent employers would be willing to practice widespread workforce SARS-CoV-2 testing. Nonetheless, for many American workers, it became a job requirement to become vaccinated or undergo serial testing while the threat of the pandemic was ongoing [12,13,14]. As employers considered making vaccinations mandatory or strongly encouraged, weekly testing was posed as an alternative safety measure [15,16]. The type and frequency of SARS-CoV-2 testing activities offered by small, medium, and large U.S. employers to their respective workforces was relatively unknown early in the pandemic. To meet this information gap, our project team, as part of a larger mixed-methods study on employer testing of SARS-CoV-2 history (ETCH), interviewed U.S. employers about their experiences and perspectives during the first year of the pandemic with offering SARS-CoV-2 testing to their workforces voluntarily. In the present article, we report on the qualitative data collected from key informant interviews with participants from U.S. companies about their SARS-CoV-2 testing experiences. 

## 2. Materials and Methods

### 2.1. Study Design

There were two phases to this study, a qualitative phase, and a quantitative phase. A mixed-methods sequential exploratory study design was used to initially characterize through qualitative data and subsequently quantifying, through survey data, facilitators and barriers for SARS-CoV-2 testing among U.S. businesses [17]. In this article, data are presented from the qualitative phase. The goals for the qualitative phase were to (1) gain an understanding of barriers to SARS-CoV-2 testing near the outset of the pandemic and (2) refine questions for use in the subsequent, chiefly closed-ended survey to be sent to a large sample of U.S. companies. Prior to participating in the interview, respondents were asked to complete a 17-item “pre-interview” survey to collect information about the company and industry setting, and the respondent’s socio-demographic characteristics. Survey results were used to describe industries and settings provided through the interviews but were not referenced at the time of the interview. This activity was reviewed by CDC and was conducted consistent with applicable federal law and CDC policy (See e.g., 45 C.F.R. part 46.102(l)(2), 21 C.F.R. part 56; 42 U.S.C. §241(d); 5 U.S.C. §552a; 44 U.S.C. §3501 et seq). 

### 2.2. Participant Recruitment and Eligibility Criteria

The project team, supported by CDC’s National Institute for Occupational Safety and Health (NIOSH), used its existing professional networks to identify company owners, upper-level managers, human resource leaders, or health and safety officers for in-depth interviews. The recruitment goal was to identify and conduct semi-structured key informant interviews with company leaders. Eligibility criteria for participation in the study were adults age ≥18 years who speak and write in English and work for, or own, a U.S. business principally operating in the United States. Company leaders of all races and ethnicities were encouraged to participate. To achieve diversity of responses across U.S. sectors, existing NIOSH National Occupational Research Agenda (NORA) networks were used to invite and conduct interviews with members from the following industry sectors: Agriculture, Forestry & Fishing (except Wildland Firefighting and including seafood processing); Construction; Healthcare & Social Assistance (including Veterinary Medicine/Animal Care); Manufacturing (except seafood processing); Mining; Oil and Gas Extraction; Public Safety (including Wildland Firefighting); Services (except Public Safety and Veterinary Medicine/Animal Care); Transportation, Warehousing & Utilities; and Wholesale and Retail Trade. Interviewees were not provided any compensation for participation in the study. 

### 2.3. Key Informant Interviews

Twenty interviews were conducted from January through April 2021 using a virtual meeting platform that allowed for audio recording and transcription of the interview [18]. Participants were instructed to leave their name blank when they logged onto the platform and to not transmit their video image to provide privacy and confidentiality in the study. Project team members followed a semi-structured interview script (Supplementary Materials), with most interviews running under 30 min. The generated transcripts were reviewed by the team member conducting the interview, and transcription errors were corrected before forwarding to other team members for thematic coding. 

### 2.4. Data Analysis of Survey Instrument and Key Informant Interviews

Frequency and descriptive statistics for all 17 items in the pre-interview survey were tabulated. Categorical variables were expressed as frequency and percent. Statistical analysis of survey data was performed using the Statistical Package for the Social Sciences (IBM SPSS Statistics for Mac, version 24.0, IBM: Armonk, NY, USA). The transcribed files were exported as documents and uploaded into MaxQDA Analytics Pro 2020 (Verbi, GmbH, Berlin, Germany), a computer-assisted qualitative data analysis program [19]. A preliminary coding scheme was created based on the semi-structured script and first completed transcripts. This was shared with the study team for review. From there, following a grounded theory approach, [20] first round coding was completed by one coder (NC), and reviewed and revised by the second coder (CP). Any discrepancy in coding was discussed by both coders until consensus was reached. The coders created memos to document the definition of code labels prior to creating open, axial, and selective code systems [21]. A statement in a transcript may be coded with more than one code (e.g., “we sent people down to the health department” could be coded for “partnerships” and “testing off-site”). Data saturation was determined by analyzing the repetition of information across respondents [22]. Other study team members reviewed tables organized by themes that contained coded segments with links to original locations in transcripts [23]. The study team provided additional interpretation and analyses of the themes and codes, collapsing 12 themes into six. 

## 3. Results

### 3.1. Pre-Interview Survey Data

Table 1 presents the breakdown of 20 interviews analyzed for this study. The interviews were well distributed across the NORA industry sectors, with over-representation of the construction sector. Most respondents were with companies that had been in business for more than 10 years (90%), and with companies that had 50 or more full- and part-time employees (85%, Table 1). Twelve (60%) of the 20 respondents indicated that their company offered some form of COVID contact tracing. 

Among the 20 participating companies, 11 (55%) offered SARS-CoV-2 testing to their employees. Of those, five (46%) used partnerships with local labs or healthcare providers for testing (Table 2).

Among the 11 companies whose representative reported providing SARS-CoV-2 testing, on average, two types of tests were offered, of which real-time reverse transcriptase polymerase chain reaction (RT-PCR) (63.6%) was the most frequent. Turn-around time for SARS-CoV-2 test results was usually three or more days (62.5%). Over the course of the pandemic, company representatives reported in the pre-interview survey using various methods for return-to-work (i.e., for workers who tested positive and those workers exposed to someone with SARS-CoV-2 infection), with some type of non-test-based strategy, such as in-person screening for SARS-CoV-2 signs/symptoms (45.5%), being a slightly more common approach than SARS-CoV-2 test-based strategies (36.4%), such as antigen or antibody tests.

### 3.2. Qualitative Interview Themes

Key informant interview respondents provided rich descriptions of the facilitators and barriers to SARS-CoV-2 testing encountered during the early months (i.e., March through August 2020) of the pandemic. Table 3 presents illustrative quotes for six main themes that contextualize the lived experiences and challenges encountered by company leaders in learning about and offering SARS-CoV-2 testing to their workers. The emerging themes were: (1) access to and knowledge of SARS-CoV-2 tests, (2) strategies for symptomatic and asymptomatic testing; (3) type and availability of personal protective equipment (PPE) to mitigate coronavirus exposures; (4) design of return-to-work policies; (5) guidance for and communication about SARS-CoV-2 testing; and (6) use of contact tracing and vaccination to limit SARS-CoV-2 risk. 

#### 3.2.1. Theme 1: Access to and Knowledge of SARS-CoV-2 Tests

Employers encountered numerous challenges in implementing SARS-CoV-2 testing, whether doing it themselves (healthcare organizations), or sending employees out for tests. The challenges can be categorized as “Initial and Resolved” versus “Ongoing,” and within those two groups, by problems related to the test/testing procedure, logistics of testing workers, employee management, or broader organizational concerns. About half of the employers have resolved many of their initial problems with locating testing facilities or vendors, accessing testing supplies, or encouraging their workers to take SARS-CoV-2 testing seriously. Respondents indicated that the most serious ongoing problems included reductions in workforce due to positive test results, waiting for test results, or quarantine and isolation protocols. This creates a concomitant burden on other staff or curtails company production or service. 

#### 3.2.2. Theme 2: Strategies for Symptomatic and Asymptomatic Testing

Some respondents indicated their respective companies were not doing, and could not do, routine testing of asymptomatic employees due to the cost, lack of interest from employees, or lack of perceived ‘value’ to the company, compared to other measures such as employee screenings and quarantine. This varied over time, with some organizations becoming more dependent on testing, whereas others discontinued the use of testing in favor of screening employees for symptoms. 

Several respondents described protocols for guiding employees to testing depending on where exposure possibly took place, i.e., at the worksite or in the community. Responsibility for testing was seen to be an employer burden if the exposure was acquired while working. Respondents indicated that employees who believed they were exposed elsewhere were asked to use their own healthcare providers and resources for SARS-CoV-2 testing. 

Many respondents described using temperature checks in the workplace at the outset of the pandemic, but retired those in favor of self-reported symptoms, some based on questionnaires. Those whose workers interacted directly with the public (e.g., education, healthcare) reported a perception of doing more routine testing than other sectors in their area. However, only one of the 20 interviewees, an educational organization, was continuing to test asymptomatic employees at the time of the survey. Such testing was considered cost-prohibitive, and the tests were not available at that scale. Several respondents described collaborations with local health departments or clinics, or the availability of on-site or nearby laboratories, which greatly facilitated testing. During the first year of the pandemic, respondents without such arrangements tended to send employees to their own healthcare providers or wherever tests were being offered (e.g., local pharmacies).

#### 3.2.3. Theme 3: Type and Availability of Personal Protective Equipment (PPE) to Mitigate Coronavirus Exposures

The perception of SARS-CoV-2 risk and exposure was greatest early in the pandemic as all companies were learning about transmission routes. As understanding of hazard and mitigation strategies improved, most respondents in this qualitative study (with notable exceptions of healthcare, some in retail, and transportation) reported that they felt their employees were at no additional risk, beyond what employees experienced in the community, of contracting SARS-CoV-2 on the job. Mitigation strategies mentioned included testing, physical distancing, cleaning, use of negative pressure rooms (healthcare), and use of PPE. There was general awareness of the higher risk associated with confined indoor spaces versus outdoor spaces, and of the importance of employee participation in developing safety and health protocols. Once protocols were put in place to deal with exposures, concern decreased over time. 

Use of PPE prior to the pandemic varied by NORA sector, with heavy industries (such as oil and gas extraction, manufacturing, coal mining, and some occupations within transportation) accustomed to protective equipment for handling hazardous materials. Other industries that routinely used PPE prior to SARS-CoV-2 included construction workers wearing hard hats and boots; grocery retail involving raw meat and cutting using cut-resistant gloves and face masks; agricultural workers using respiratory protection for grain dust and pesticides; and health and safety workers accustomed to masks and gloves. Many stated that while such protections were common on the job, the idea of wearing masks outside of work was new. Some study participants described how SARS-CoV-2 transformed the use of PPE, and this factor seemed relatively unimportant in decisions pertaining to testing except in the healthcare area, where PPE was thought to prevent transmission of SARS-CoV-2 from potentially infected workers to patients, and vice versa. 

#### 3.2.4. Theme 4: Design of Return-to-Work Policies

U.S. companies employed varying guidelines for SARS-CoV-2 return-to-work (RTW), for workers who tested positive, and workers exposed to a positive individual, based on the industry and sources of information guiding RTW policies. Eighty percent of respondents required a negative test initially until this was perceived as impractical due to the long wait periods to receive results, or they doubted the validity of test results (i.e., employees would test positive for long periods of time when they no longer showed symptoms, or vice versa). Respondents from healthcare sectors reported having employees returning to work while testing positive (if symptom free) as the company leadership felt their PPE prevented transmission. Most interviewees reported that they stopped requiring employees to be symptom-free (particularly no fever) for three days following a period of quarantine after possible exposure to COVID-19, which they then reported moving from 14 to 10 to seven days. Some mentioned this was following quarantine reductions issued by the CDC. Some respondents in this study reported that there was division between those believing employees took advantage of test results and quarantine guidelines to have paid time off, and others (mostly those using hourly workers) who were concerned that employees would not get tested if it meant they had to miss work, i.e., they would come to work SARS-CoV-2-positive or sick rather than miss a day’s pay. Respondents indicated there were clear tensions between the operational and production needs of companies and the limits set on time away from work for those who tested positive or who came into contact with positive workers. 

#### 3.2.5. Theme 5: Guidance and Communication of SARS-CoV-2 Testing

Most respondents claimed to follow the guidance from either the CDC or their state/local health department. However, they had different interpretations of the same guidance. Respondents from two larger organizations mentioned the need to adhere to guidance from multiple states or counties, and this made management more difficult. Several respondents did not mention any clear sources of guidance. Others reported difficulties following guidelines, either because guidelines were unclear, were perceived as conflicting or changing rapidly, or did not meet their needs or facilitate their processes. 

Respondents indicated that their companies experienced a wide variation in the amount of information that was communicated to them by federal, state, and local governmental bodies about testing and vaccines. Not all employers communicated about both; some communicated about testing but not vaccines, and vice versa. Several participants reported discrepancies between federal and state recommendations which resulted in confusing communications from the employers. One respondent suggested involving the health department or medical personnel in helping organizations interpret guidance and communicate it more effectively.

#### 3.2.6. Theme 6: Use of Contact Tracing and Vaccination to Limit SARS-CoV-2 Risk

A related theme that emerged from the interviews on SARS-CoV-2 testing was contact tracing. Twelve of 20 respondents said their company was doing some form of contact tracing, and one was unsure what the company was doing. Others described procedures ranging from informal notification (i.e., co-workers contacting other co-workers, outside of a formal contact tracing program) to descriptions equivalent to the definition provided by the interviewer (i.e., tracing contacts of persons with SARS-CoV-2 infection); three company representatives mentioned more formal methods of notification, but lack of referrals or long-term follow-up. There were no reasons provided for not doing tracing; it was merely said that it did not seem necessary (e.g., if the company was primarily in a work-from-home state), or that it was turned over to the health department. Eight of 20 respondents indicated tracing beyond employees or suggested strong collaborations with health departments. Interviewees indicated that responsibility for contact tracing by a company seemed to depend on whether the primary exposure was thought to have occurred at work (yes, employer responsible) or elsewhere (no, employer not responsible, even though the employee might be out sick, and others might have interacted with him/her in a non-work setting).

Some interviewees mentioned challenges with the implementation of SARS-CoV-2 vaccination within their companies, efforts to conduct SARS-CoV-2 vaccination campaigns, and barriers to scheduling appointments to get vaccinated. These interviewees, similar to other participating companies, reported that finding dates and times to release employees for vaccination at sites located at the county- or city-level was challenging. 

## 4. Discussion

Several factors, both facilitators of, and barriers to, SARS-CoV-2 testing, were identified in this initial qualitative phase of the ETCH study. Since the start of the SARS-CoV-2 pandemic, U.S. companies have struggled with access to and understanding of information about SARS-CoV-2 testing. This study documented that the company leaders interviewed were confused about reliable and consistent sources of SARS-CoV-2 information. Respondents also perceived conflicting information about SARS-CoV-2 testing requirements between local and federal guidelines. These findings are consistent with research by McElfish et al., who found unclear guidance and uncertainty regarding SARS-CoV-2 testing guidelines among residents from the State of Arkansas [24]. Company owners in the same communities where their employees reside were receiving mixed messages about where to access and how to interpret results of SARS-CoV-2 testing. 

We found that participating company leaders were challenged by testing symptomatic and asymptomatic individuals, particularly in companies with larger workforce sizes. Some U.S. sectors and companies, during the study observation period, sought to devise strategies for testing and return to work to maintain a functional workforce. Our study findings are similar to those reported by Lecouturier et al. in a qualitative study of the general public, including working and non-working adults [25]. They found that working adults were confused about the interpretation of positive and negative SARS-CoV-2 testing results and how their testing results impacted their ability to return to work.

The current study had limitations. We had a very small sample of U.S. companies reporting on their challenges and approaches to SARS-CoV-2 testing. Our recruitment approach used the NORA sectors network, which supported the inclusion of a diversity of companies, but there are also likely variations in the facilitators and barriers to SARS-CoV-2 testing by size of company and geography. Saturation (repetition of similar responses) was achieved in this sample of interviewees, supporting a transition to the subsequent phase of this study to identify SARS-CoV-2 testing factors that could be quantified in a survey with a larger, more diverse sample of U.S. companies. Not all interviewees were owners of the company. Respondents included human resource leaders and medical, health, and safety officers who may not have had full awareness of their company’s challenges in SARS-CoV-2 testing. Additional limitations include that a majority of participating companies had been operational for over 10 years and that most participating companies had a workforce size of 50 or more employees, limiting perspectives from newer and smaller businesses. Nonetheless, this non-probabilistic sample included some responses across all employee workforce size ranges. Lastly, the responses in this pilot study are not generalizable to all U.S. industries and should not be interpreted to provide generalizable information on factors that impact SARS-CoV-2 testing among U.S. companies. Despite these limitations, this pilot study had several strengths, including a diversity of responses across U.S. industry sectors that allowed for the identification of several factors affecting use of SARS-CoV-2 testing. Using a key informant interview format allowed for the in-depth exploration of perspectives and responses from company leadership on factors, processes, and information sources that hindered or supported their businesses’ ability to offer testing and contact tracing.

## 5. Conclusions

U.S. companies have experienced several challenges since the start of the SARS-CoV-2 pandemic with offering regular and consistent SARS-CoV-2 testing. We identified six major themes about SARS-CoV-2 testing that spanned access to and knowledge of SARS-CoV-2 testing, strategies for symptom-based testing, availability of PPE, RTW policies, authoritative guidance on SARS-CoV-2 testing, as well as how contact tracing and emerging SARS-CoV-2 vaccination options would impact SARS-CoV-2 testing. This study sheds light on potentially modifiable and non-modifiable factors that could inform SARS-CoV-2 testing strategies for U.S. companies. Future studies might be able to quantify these identified themes in a larger sample of U.S. companies to inform the prioritization of factors to support U.S. businesses by sector and size in offering SARS-CoV-2 testing to their employees. 

## Figures and Tables

**Table 1:** Organization/company characteristics and SARS-CoV-2 Testing Practices among U.S. companies participating in the Employer Testing of SARS-CoV-2 History (ETCH) Phase 1 Study, January–April 2021 (*n* = 20).

Organization Characteristics	*n* (%) ^†^
**NORA Industry Sector**	
Agriculture, Forestry & Fishing (except Wildland Firefighting and Seafood Processing)	2 (10.0)
Construction	4 (20.0)
Healthcare & Social Assistance (including Veterinary Medicine/Animal Care)	2 (10.0)
Manufacturing (except Seafood Processing)	2 (10.0)
Mining (except Oil and Gas Extraction)	2 (10.0)
Oil and Gas Extraction	2 (10.0)
Public Safety (including Wildland Firefighting)	2 (10.0)
Services (except Public Safety and Veterinary Medicine/Animal Care)	1 (5.0)
Transportation, Warehousing & Utilities	1 (5.0)
Wholesale and Retail Trade	2 (10.0)
**Time Company has been Operational**	
<1 year	0 (0.0)
1–2 years	0 (0.0)
3–5 years	2 (10.0)
6–10 years	0 (0.0)
>10 years	18 (90.0)
**Total Number of Employees (full- and part-time) at Company**	
<10	1 (5.0)
10–49	2 (10.0)
50–249	8 (40.0)
250–999	3 (15.0)
>1000	6 (30.0)
**Company Offered Contact Tracing**	
Yes	12 (60.0)
No	7 (35.0)
Don’t Know	1 (5.0)

^†^ Differences in sub-total population sample due to item non-response or missing.

**Table 2:** SARS-CoV-2 testing practices and return-to-work strategies among participating companies who offered testing (*n* = 11) in the Employer Testing of SARS-CoV-2 History (ETCH) Phase 1 Study, January–April 2021.

Organization Characteristics	*n* (%) ^†, ‡^
**Return to Work Methods Used (all that apply)**	
Temperature Checks at Entry	9 (40.9)
Online/Phone App for SARS-CoV-2 Symptom Reporting	4 (18.2)
In-Person Screening for SARS-CoV-2 Signs/Symptoms	10 (45.5)
Laboratory Testing (RT-PCR, antigen, other (e.g., antibody) test)	8 (36.4)
No Special Method Beyond Self-report of Illness to Supervisor	10 (45.5)
**Primary Method to Offer SARS-CoV-2 Testing and/or Antibody Screening to Employees**	
Partnership with Local Lab or Healthcare Provider	5 (45.5)
Sent Employee to Local Health Department	3 (27.2)
Company Administers SARS-CoV-2 Testing Themselves	3 (27.2)
**Type of SARS-CoV-2 Testing Offered to Employees (all that apply)**	
Antigen testing	4 (36.4)
RT-PCR testing	7 (63.6)
Antibody testing	5 (45.5)
Not sure	1 (9.1)
**Turn Around Time for SARS-CoV-2 Tests Ordered**	
<1 day	1 (12.5)
1 day	1 (12.5)
2 days	1 (12.5)
3 days	4 (50.0)
Approximately 1 week	1 (12.5)

^†^ Among the 20 participating companies, 11 offered some type of SARS-CoV-2 testing and used some type of return-to-work strategy; ^‡^ Differences in sub-total population sample due to item non-response or missing.

**Table 3:** Themes and additional supporting quotations from U.S. companies participating in the Employer Testing of SARS-CoV-2 History (ETCH) Phase 1 Study, January–March 2021 (*n* = 20).

Theme	Code	Supporting Quotation (Participant Number)
Access and Knowledge of SARS-CoV-2 Tests	1.1—Limited access to testing supplies and training	“From the get-go, there was a shortage in testing.”—Manufacturing Respondent 5 “We have [enough tests] now for our patients, but we’ve never had enough for regular testing of our staff… PCR … becomes prohibitively expensive and not fast enough turnaround to be functional… we only have enough to do testing of symptomatic people.”—Healthcare Respondent 6
	1.2—Lack of knowledge concerning SARS-CoV-2 testing	*“*We don’t do [testing] at our store … because I don’t know how we would do that from a medical capacity. I don’t think we have the resources to do [testing].”*—*Retail Respondent 10 “I think it’s just the lack of knowledge and communication. You hear from so many different entities and it seems like nobody is telling you the same thing.”*—*Construction Respondent 20
	1.3—About half of the organizations have improved their access to and knowledge of SARS-CoV-2	“Having access to the lab really made [testing] even easier.”—Public Safety Respondent 13 “Things matured as we began to know more about the disease as testing began to become prolific in our area.”—Manufacturing Respondent 18
Strategies for Symptomatic and Asymptomatic Testing	2.1—Companies tested employees on a case-by-case basis, self-reported symptoms and upon high-risk work-place exposure events.	“We’re not … testing everybody at a certain timeframe.... But if [employees] have any … exposures, whether at work or at home, or [if there’s] any symptoms that [might] suggest COVID, then we’re testing.”—Healthcare Respondent 2 “We’ve only tested... based on exposure [and] based on symptoms.”—Healthcare Respondent 6
	2.2—Companies without on-site testing relied on the local department of health and partnerships with providers and local clinics for testing.	“We also partner with a local health care provider that, upon symptoms or an exposure, will test our personnel.”—Public Safety Respondent 4 “We have advised them to get tested on their own with their treating doctor.”—Manufacturing Respondent 11
	2.3—More than half (*n* = 12) did not conduct routine asymptomatic testing	Lack of interest from employees: “They are coal miners. Some of them would refuse to [get tested] unless you force them to. Simply because they… don’t think there’s really a big problem with [COVID] or believing in it.”—Mining Respondent 15
Type and Availability of PPE to Mitigate Coronavirus Exposures	3.1—Perception of Risk of Exposure	“I think that’s been unique in our area of work we pretty much always relied on PPE for most infection.”—Healthcare Respondent 2
	3.2—PPE was thought to prevent transmission of SARS-CoV-2 in the workplace	“They’re afraid they’re going to get sick. And we have protective barriers, and we have masks and face shields and we’re requiring employees to wear those. And we’re requiring customers to wear them.”—Retail Respondent 10 “All employees are required to wear a facial mask inside any building.”—Manufacturing Respondent 8
Design of Return-to-Work Policies	4.1—Return to Work challenges and practices	“No, we do not [require a test], we don’t test [employees but] we have a protocol as far as if you’re positive… or if you have the symptoms and you go for testing, and you’re positive… then the health department gets [involved] and says when they can come back [to work]. But not all employees want to go get a test, or you know, for whatever reason.”—Retail Respondent 7 “We decided to not require a retest prior to employees showing back up in the workplace. I think if I had to answer that it’s because there is a possibility that they could still test positive. It also makes sense, going with public health guidelines, [that] after they completed their 10 days and they’ve been symptom free for at least two days, they are eligible to come back to the workplace.”—Education Respondent 12 “So as far as the return-to-work process, they were offered the opportunity to go get testing and if they accept then we send, and that was an antibody test. We did that for three months, but that was the only time we did that.”—Manufacturing Respondent 5
Guidance and Communication of SARS-CoV-2 Testing	5.1—Guidance from local/state/federal authorities	“There’s a lot of communication goes out to all of the employees, including people that work from home, if they have any of the symptoms they contact their designated medical department.”—Manufacturing Respondent 5 “Lack of Communication when a person should be tested, how often they should be tested.... After exposure? or should it be routine?”—Public Safety Respondent 13
Use of Contact Tracing and Vaccination to Limit SARS-CoV-2 Risk	6.1—Contact tracing	“They did contact trace related to that [work-related exposure]. But it was a very limited scope because the buildings were already shut down [when it happened].”—Education Respondent 1 “We will ask the question where do you think you came in contact with someone? And they will give us, you know my spouse is positive or my child is positive from school and then we would give that information on to the local public health group.”—Public Safety Respondent 4
	6.2—Vaccination	“We’re trying, as leaders in our departments to talk about getting vaccinated.”—Healthcare Respondent 6 “The platform that was created for the testing to schedule appointments for employees to be tested has been able to be utilized as well for the vaccinations.”—Public Safety Respondent 13

## Data Availability

Data will be available from NIOSH when objectives of the research are complete. Please contact aeastlake@cdc.gov.

## Supplementary Materials

The following supporting information can be downloaded at: https://www.mdpi.com/article/10.3390/ijerph191811805/s1, Employer Testing of COVID-19 History (ETCH) Study Focus Group Discussion/Key Informant Interview Script.

## References

[1] Honey-Rosés J., Anguelovski I., Chireh V.K., Daher C., Konijnendijk van den Bosch C., Litt J.S., Mawani V., McCall M.K., Orellana A., Oscilowicz E. (2020). The impact of COVID-19 on public space: An early review of the emerging questions–design, perceptions and inequities. Cities Health.

[2] Waltenburg M.A., Victoroff T., Rose C.E., Butterfield M., Jervis R.H., Fedak K.M., Gabel J.A., Feldpausch A., Dunne E.M., Austin C. (2020). Update: COVID-19 among workers in meat and poultry processing facilities―United States, April–May 2020. Morb. Mortal. Wkly. Rep..

[3] Skoll D., Miller J.C., Saxon L.A. (2020). COVID-19 testing and infection surveillance: Is a combined digital contact tracing and mass testing solution feasible in the United States?. Cardiovasc. Digit. Health.

[4] Zhang T., Gerlowski D., Acs Z. (2021). Working from home: Small business performance and the COVID-19 pandemic. Small Bus. Econ..

[5] Akpan I.J., Udoh E.A.P., Adebisi B. (2020). Small business awareness and adoption of state-of-the-art technologies in emerging and developing markets, and lessons from the COVID-19 pandemic. J. Small Bus. Entrep..

[6] Manabe Y.C., Sharfstein J.S., Armstrong K. (2020). The need for more and better testing for COVID-19. JAMA.

[7] King J.S. (2020). Covid-19 and the need for health care reform. N. Engl. J. Med..

[8] Santellano K.J.E., Studies R. (2021). Compounded inequality: How the US Paycheck Protection Program is failing Los Angeles Latino small businesses. Ethn. Racial Stud..

[9] Fairlie R., Fossen F.M. (2021). Did the Paycheck Protection Program and Economic Injury Disaster Loan Program get disbursed to minority communities in the early stages of COVID-19?. Small Bus. Econ..

[10] White House Centers for Disease Control and Prevention. Guidelines: Opening up America again. https://www.whitehouse.gov/openingamerica/-criteria.

[11] Center for Medicare & Medicaid Services Opening up America again: Centers for Medicare & Medicaid Services (CMS) Recommendations Re-Opening Facilities to Provide Non-Emergent Non-COVID-19 Healthcare. https://www.cms.gov/files/document/covid-flexibility-reopen-essential-non-covid-services.pdf.

[12] Emanuel E.J., Skorton D.J.J. (2021). Mandating COVID-19 Vaccination for Health Care Workers. Ann. Int. Med..

[13] Rothstein M.A., Parmet W.E., Reiss D.R.J. (2021). Employer-Mandated Vaccination for COVID-19. Am. J. Public Health.

[14] Gostin L.O., Parmet W.E., Rosenbaum S.J.J. (2022). The US Supreme Court’s Rulings on Large Business and Health Care Worker Vaccine Mandates: Ramifications for the COVID-19 Response and the Future of Federal Public Health Protection. JAMA.

[15] US Equal Employment Opportunity Commission What you should know about COVID-19 and the ADA, the Rehabilitation Act, and other EEO laws. https://www.eeoc.gov/wysk/what-you-should-know-about-covid-19-and-ada-rehabilitation-act-and-other-eeo-laws,.

[16] Schulte P.A., Piacentino J.D., Weissman D.N., de Perio M.A., Chiu S.K., Radonovich L.J., Trout D., Beezhold D., Hearl F.J., Howard J. (2021). Proposed Framework for Considering SARS-CoV-2 Antigen Testing of Unexposed Asymptomatic Workers in Selected Workplaces. J. Occup. Environ. Med..

[17] Creswell J.W., Creswell J.D. (2017). Research Design: Qualitative, Quantitative, and Mixed Methods Approaches.

[18] Archibald M.M., Ambagtsheer R.C., Casey M.G., Lawless M. (2019). Using zoom videoconferencing for qualitative data collection: Perceptions and experiences of researchers and participants. Int. J. Qual. Methods.

[19] Kuckartz U., Rädiker S. (2019). Analyzing Qualitative Data with MAXQDA.

[20] Charmaz K. (2014). Grounded theory in global perspective: Reviews by international researchers. Qual. Inq..

[21] Strauss A., Corbin J.M. (1997). Grounded Theory in Practice.

[22] Glaser B.G., Strauss A.L. (2017). Discovery of Grounded Theory: Strategies for Qualitative Research.

[23] Watkins D.C. (2017). Rapid and rigorous qualitative data analysis: The “RADaR” technique for applied research. Int. J. Qual. Methods.

[24] McElfish P.A., Cleek A.B., Willis D.E., Purvis R.S., James L.P. (2021). Leveraging community engagement capacity to address COVID-19 disparities among Pacific Islander and Latinx Communities in Arkansas. J. Clin. Transl. Sci..

[25] Lecouturier J., Kelly M.P., Graham F., Meyer C., Tang M.Y., Goffe L., Bonell C., Michie S., Sniehotta F.F. (2021). Public understanding of COVID-19 antibody testing and test results: A qualitative study conducted in the U.K. early in the pandemic. Soc. Sci. Med..

