# Supplementary material for: Barriers to SARS-CoV-2 Testing among U.S. Employers in the COVID-19 Pandemic: A Qualitative Analysis Conducted January through April 2021
Source: Int J Environ Res Public Health. 2022 Sep 19;19(18):11805. doi: 10.3390/ijerph191811805 (PMC9517542; doi:10.3390/ijerph191811805)
Supplement: Supplementary file 1 [file ijerph-19-11805-s001.zip › ijerph-1869805-supplementary.pdf]

SUPPLEMENTARY MATERIAL

Employer Testing of COVID-19 History (ETCH) Study
FOCUS GROUP DISCUSSION/ KEY INFORMANT INTERVIEW SCRIPT

TELL ME ABOUT YOUR COMPANY

- Tell me generally about your company. What type services or products do you offer/produce?

- How is your company organized in terms of work locations and workforce?
    - Probe: Changes due to COVID-19
    - Probe: Do all of your employees work in the same building?

- Under normal circumstances, do your employees use any kind of protective equipment?

- Under normal circumstances, do you have a worksite health or wellness program for employees? (If yes, please describe)

- Have you made any modifications due to COVID-19?

TELL ME ABOUT COVID-19 TESTING AT YOUR COMPANY

- Are you actively testing your employees for COVID-19? [If no, skip to …..]
    - Did you partner or collaborate with a company for COVID-19 testing? Onsite or offsite?
    - Any engagement with local health department for testing and how?
    - Encourage testing through healthcare and/or primary care physician?

- How do you navigate deciding when and how to test employees?

- Describe **safety or health hazards** you thought about in terms of COVID-19 testing your workers.
    - How do you identify these safety/health hazards?
    - How do you prevent and protect your workers from these hazards?
    - When exposed to these hazards, how do you deal with them?
    - What support, if any, should be provided to protect the health and safety of *your workers from COVID-19?*

- Do you think your workers are at an increased risk for illnesses or injuries from COVID-19?
    - If yes, what kind of illnesses?

        - If yes, what kind of injuries?

- Describe how the health and safety risks of COVID-19 exposure are different for your employees compared to other type of occupations and/or companies.

- What are the main challenges you face to COVID-19 test your worker?

- What sources does your company use for COVID-19 testing and screening?
    - What guidance for testing?
    - What guidance for screening?

- What would be required for your company to implement routine testing of asymptomatic employees?
    - What are the barriers? (cost, time, access to technology, knowledge, etc)

- Have you implemented a contact tracing program?
    - How did it go?
    - What worked and what did not work?
    - Who is responsible for conducting contact tracing?

- Do you have a return-to-work policy following COVID-19 infection?
    - What does it involve? Examples: checking for signs/symptoms (e.g., fever, cough, shortness of breath, abnormal taste/smell) or laboratory test.
    - If you don't use a lab test, what are some of the reasons?

Conclusion:

Are there any areas we did not talk about that you think are important for us to know?

[If researchers have brief clarifying questions, they will ask.]

Thank you for participating. We learned a lot and it was great to talk to you. We really appreciate that you have shared your thoughts with us.
